# Enterosorption may contribute to the reactivation of anticancer immunity and be an effective approach to tumor growth control

**DOI:** 10.3389/fimmu.2024.1366894

**Published:** 2024-02-26

**Authors:** Valentin P. Shichkin

**Affiliations:** OmniFarma LLC, Kyiv, Ukraine

**Keywords:** immunity, cancer, gut microbiota, enterosorption, detoxification

## Introduction

**Figure 1:**
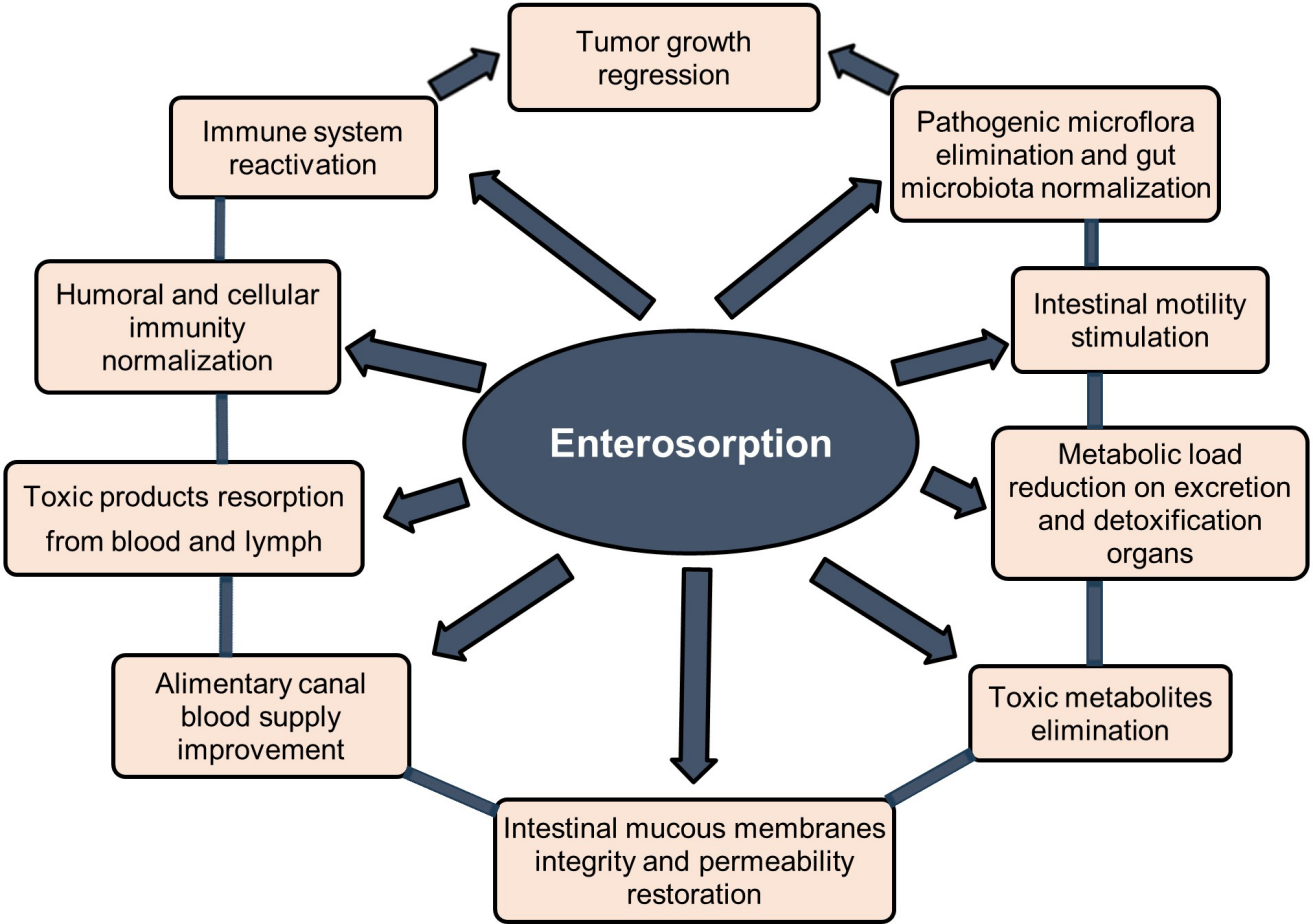
Potential clinical effects of enterosorption in cancer patients. Modified from Shichkin et al., 2023 (3); this is an open-access article distributed under the terms of the Creative Commons Attribution License (CC BY).

Enterosorption is one of the safest and most effective methods for binding and excretion of various exogenic and endogenic toxins and metabolites from the body based on oral administration of an enterosorbent that can absorb toxic substances in the lumen of the gastrointestinal tract without entering into chemical reactions. Enterosorbents can provide body detoxification by several mechanisms - directly in the gut and by resorption from blood and lymph (1–3). Many studies show the high efficiency of enterosorption detoxification in the complex treatment of various diseases including cancer, allergy, dysbiosis, hepatobiliary toxicosis, and various systemic intoxications (1–7). Due to the selective adsorption of toxins and pathogenic microflora by enterosorbents the gut microbiota and digestive processes are normalized, and the condition of the intestinal mucosa, gastrointestinal tract, hepatobiliary, and immune system is significantly improved. This complex effect of enterosorption contributes to the rapid relief of clinical symptoms and improves the disease prognosis (1–7) (**Figure 1**).

Low invasiveness and the absence of pronounced side effects provide additional grade benefits of enterosorbents use for body detoxification both during tumor growth and treatment normalizing gut microbiota and reducing tumor-induced immunosuppression (4–7) and thus improving conditions for antitumor immune response. Arguments in favor of this are some experimental data indicating that the immune system may respond to tumor antigens during acute inflammatory reactions leading to tumor regression. The acute inflammatory response is the first line of defense promoting innate and adaptive immune responses. However, in the case of prolonged acute inflammatory reaction, it could be transformed into chronic inflammation resulting in an immunosuppressive microenvironment (8). These changes promote the activation of oncogenes, the damage of DNA and protein, the release of ROS, and affect multiple signaling pathways including STAT3, K-RAS, NF-κB, and P53 supporting tumor growth (8–10).

Cancer cells use a variety of mechanisms allowing them to evade effective control by the immune system, leading to accelerated tumor growth and a fatal outcome. One of the main mechanisms by which a tumor gains undeniable advantages in confrontation with the immune system may be the development of metabolic stress due to the accumulation of toxic products in the blood and organs, both actively produced by tumor cells and resulting from their death (7, 11, 12). The result of this may be systemic immunosuppression, the loss of the ability to recognize tumor antigens and provide any effective resistance to its growth (7, 11). This condition is known as tumor-specific T-cell anergy (10, 13) or more generally, immunological tolerance (10, 14).

It can be assumed that the blockade of immune recognition *in vivo* following cancer extends not only to tumor antigens but also to various bacteria, both endogenous and exogenous. This especially may be evident at the final stage of tumor growth, shortly before lethal as it was shown in the mouse experimental brain tumor model (11). At the same time, immune cells extracted from mouse blood and lymphoid organs exhibited high immunocompetence concerning the cancer cells *in vitro* (11).

While T-cell tolerance to tumors remains a major barrier in cancer immunotherapy, combined strategies using vaccination together with agonists of co-stimulatory pathways and inhibitors of immunologic checkpoints are capable of overcoming tolerance and generating significant anti-tumor response (8–10, 12, 15–18). In particular, studies in genetically manipulated mice showed that various immune system components can modify or even eliminate carcinogen-induced and spontaneously arising cancers (9, 15, 16). This may suggest that, probably, the tumor is not always the direct cause of death. In certain cases, this may be due to the accelerated growth of pathogenic microflora (19–21) or even following the dysbiosis decreasing the diversity of normal microbiota and leading to abnormal growth of endogenous opportunistic microflora due to loss of control by the immune system (22, 23) that may lead to fatal consequences much faster than would be expected from tumor growth itself.

**Figure 2:**
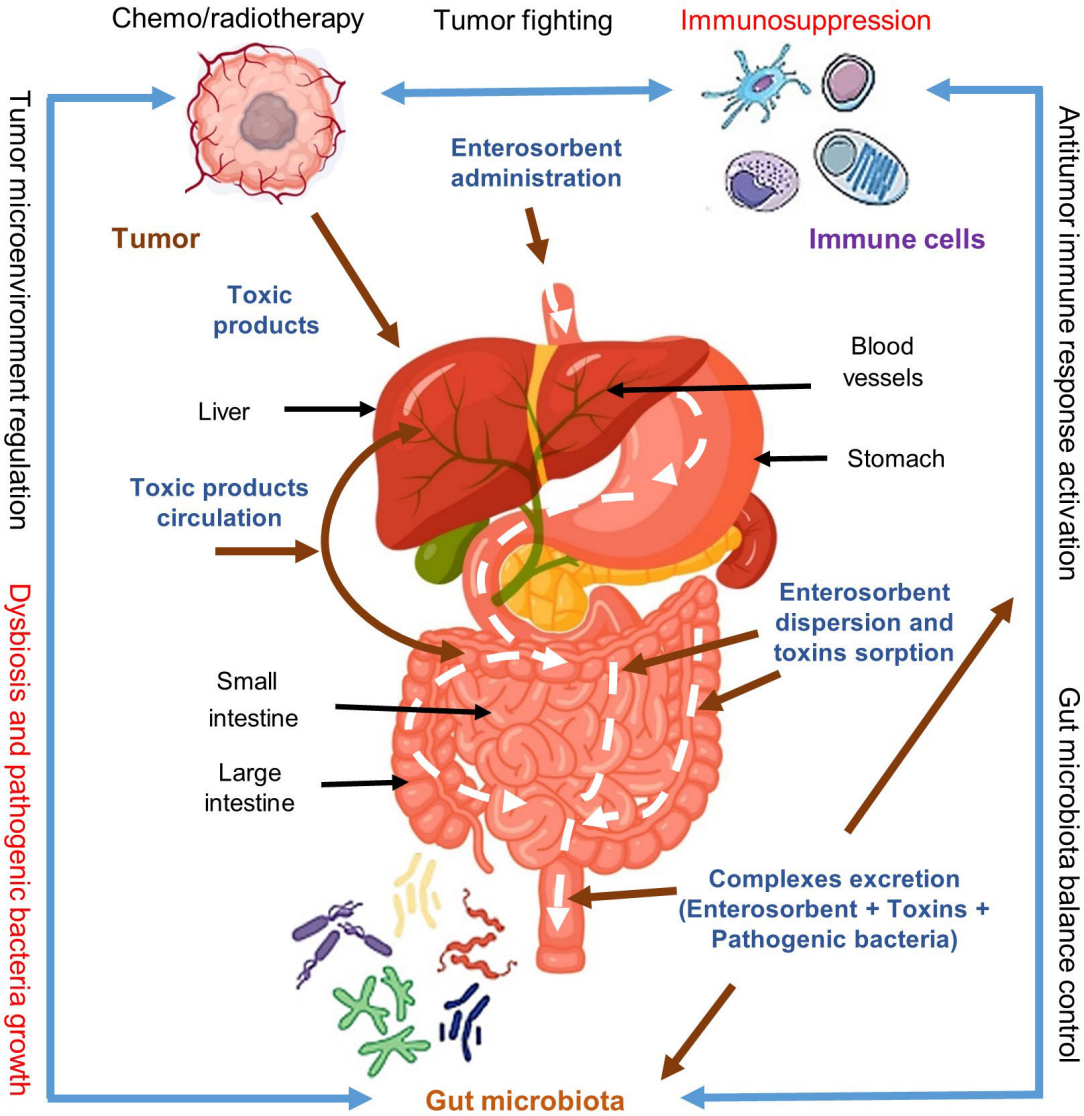
Impact of enterosorption on the cancer-gut microbiota-immune system axis interaction and cancer treatment. Cancer cells produce toxic immunosuppressive metabolites and actively form a safe-for-yourself microenvironment recruiting for this also some species of gut microbiota and thus escaping the immune system control. Applied chemo/radiotherapy for cancer treatment increases the immunosuppression and gut microbiota dysbiosis providing conditions for uncontrolled growth of pathogenic microflora and cancer relapses. Enterosorption has no direct effect on cancer cells however it may effectively remove from the body the toxic tumor products and other harmful components arising due to chemo/radiotherapy as well as help remove pathogenic microflora and restore normal gut microbiota. These complex effects of enterosorption may result in the reactivation of the immune system and increase anticancer immunity. Modified from Shichkin et al., 2023 (3); this is an open-access article distributed under the terms of the Creative Commons Attribution License (CC BY).

Following these assumptions, it can be expected that the normalization of gut microbiota and detoxification of the body with the help of enterosorbents, especially in late cancer stages and especially in the context of chemo/radiotherapy courses may significantly improve the patient’s state and recruit immune system to effective anticancer response both in the process of cancer treatment and for preventing possible relapses (**Figure 2**). This opinion, in particular, is supported by a set of publications from Ukrainian researchers (4–7). However, the assumption that pathogenic microflora may be indeed often the immediate and leading cause of death in cancer patients is hypothetical and should be investigated.

## Impact of enterosorption on the cancer-immunity axis

The primary line of anti-tumor defense is components of the innate immune system including natural killer cells (NK cells), macrophages, neutrophils, and innate lymphoid cells (ILCs), as well as pro-inflammatory mediators and cytokines (leukotrienes, prostaglandins, tumor necrosis factor, interleukins 1, 6, and others) (8). The formation of the primary tumor focus attracts innate immune cells leading to the development of an acute inflammatory reaction with two possible subsequent outcomes.

The optimistic scenario concludes with the destruction of the tumor focus with involvement in later stages elements of the adaptive immunity in this process.

The pessimistic scenario results in the transition of the acute protective inflammatory reaction into a chronic form. The microenvironment created by the tumor during this process promotes its progression and accumulating toxic metabolites and biologically active products block the activity of the innate immunity and adaptive immune response forming immune tolerance to tumor antigens (8, 12). Nevertheless, as suggested by some experimental data this tolerance is not absolute and can be reversed either by removing the toxic blockade (11) or through modern technologies regulating antitumor T-cell activity by inhibiting the STAT3 pathway in tumor cells since STAT3 activity and increased levels are associated with poor cancer prognosis in patients (10, 15, 17). More recent approaches employ immune checkpoint inhibitors targeting cytotoxic T-lymphocyte antigen 4 (CTLA4-4), programmed cell death protein 1/programmed death-ligand 1 (PD-1/PD-L1) and lymphocyte activation gene-3 (LAG-3) in T- cell signaling pathways (12, 18).

This circumstance opens a window of opportunities for reactivating the immune system through active detoxification of the body from cancer metabolites by enterosorbents (**Figure 2**). Therefore, enterosorption may serve as a potential strategy to break the cycle of chronic inflammation and immune tolerance induced by the tumor microenvironment eliminating toxic metabolites and harmful biologically active products and restoring the innate and adaptive antitumor immune responses.

## Impact of enterosorption on the cancer-gut microbiota-immunity axis

It has long been known that cell wall components of bacteria have adjuvant properties in the formation of adaptive immune response to foreign antigens and an acute inflammatory reaction provoked by intestinal microflora can lead to spontaneous regression of some immunogenic tumors (19). However, the process of interaction between microbiota and tumors is complex and contradictory. Microbiota can actively influence the formation of the tumor microenvironment, be an important part of this microenvironment, directly invade the tumors and modify tumor cell biology, and regulate the local immunity thus stimulating regression or, conversely, tumor growth, and directly affecting the treatment efficacy (**Figure 2**). Some gut bacteria can induce DNA damage or modify DNA repair mechanisms contributing to cancer initiation and promotion (24–35). Therefore, the final result depends on many factors, among which both the bacteria strains and the type and localization of the tumors, as well as the state of the immune system as a whole and its local activity are important.

When global systemic immunosuppression or immunological tolerance is formed as a result of the accumulation in the body of toxic biologically active tumor products this can lead to uncontrolled growth of pathogenic and opportunistic gut microbiota which also becomes pathogenic. In turn, this pathogenic microbiota may promote both the acceleration of tumor growth and the further increase the local and systemic immunosuppression and tumor-specific tolerance (19–24, 32, 35).

In the past decade, the relationship between gut microbiota and the immune system has gained paramount importance. There is now essential evidence that the state of gut microbiota plays a direct and crucial role in the formation and regulation of antitumor immune response and disbalance in the composition of gut microbiota leads to adverse consequences in both innate and adaptive immune response shaping (19, 24, 29–31, 33). In particular, recent preclinical and clinical studies suggest that the intestinal microbiota and its metabolites affect cancer patients’ response to immunotherapy by PD-1/PD-L1 and CTLA-4 immune checkpoint inhibitors (24).

Normalization of gut microbiota exerts a positive immunomodulatory effect on innate and adaptive immunity through diverse mechanisms. This immunomodulatory effect of gut microbiota is not confined solely to the local level within the gastrointestinal tract but extends to the systemic level, impacting immune components of the blood, lymph, and peripheral lymphoid tissues and organs. Moreover, there is even the possibility that bioactive microbial metabolites influence the processes of immune maturation in central organs of the immune system such as the bone marrow and thymus affecting the key immunoregulatory pathways (19, 24, 29–31, 33).

Based on these data, the normalization of gut microbiota and body detoxification becomes of paramount importance in shaping an adequate immune response not only to tumor antigens but also to a broad spectrum of other harmful substances including pathogenic microflora and allergens (3). In this context, enterosorption may emerge as a crucial additional factor in restoring compromised immune competence (**Figures 1**, **2**).

## Impact of enterosorption on cancer treatment

While modern approaches for cancer treatment, like immunotherapy, are actively employed in current strategies (10, 15–19), conventional treatments (chemo/radiotherapy) still keep their leading positions in routine oncological practice. However, since the immune system is very sensitive to these treatments, they have dramatic damage sequences for the immunity leading to deep immunodeficiency and following risk of cancer relapses, dysbiosis, and pathogenic infections.

There are at least several risk factors that contribute to this global challenge. One of them is direct damage to the lymphoid organs resulting in the generation of toxins from both destroyed lymphoid tissues and tumors. These toxins potentially may be modifying factors increasing tumor-specific tolerance and systemic immunosuppression.

Other challenges are connected with gut microbiota disbalances and uncontrolled growth of pathogenic microflora (20, 21) promoted by immunodeficiency arising following tumor growth (24, 25) and chemo/radiotherapy (4–7). These treatments lead to not only in increasing of immunodeficiency but also potentially may promote the formation of immunological tolerance to pathogenic microflora.

While enterosorbents have already proven to be effective in detoxification for dysbiosis, alcohol intoxication, industrial toxin poisonings, and food allergies (1–3), studies regarding their application in various forms of oncological diseases still are scarce and are limited to local preclinical investigations with use of carbon adsorption therapy for Lewis lung carcinoma, Geren’s carcinoma, radiation-induced breast cancer, and acute radiation sickness and iatrogenic leukopenia (4–7). These studies demonstrated the positive effects of enterosorption on the restoration of homeostasis, normalization of biochemical and hematological blood parameters, a significant reduction in the toxic load caused by the use of chemotherapy and radiation, as well as an increase in the survival rate of experimental animals.

Therefore, enterosorbents possess several properties that make them potentially effective adjunctive tools in combating cancer. At the very least, they could significantly alleviate the patient’s condition during chemo/radiotherapy, accelerating the recovery of the immune system after such treatment courses through effective detoxification (4–7). Additionally, selective nonspecific absorption of pathogenic microflora and their toxic metabolites by enterosorbents may preserve cancer patients from the fatal infection process (**Figure 2**). At this, the risks of side effects associated with enterosorbent use are minimal or absent when appropriate recommendations are followed (3).

## Discussion

The immunosuppressive effect of tumor growth is brought about by the comprehensive action of many harmful components, among them are the products of tumor metabolism, the breakdown of the tumor itself, as well as toxic metabolites of pathogenic microflora (8, 10, 19–31). These components may directly damage the cell compartments of the immune system or modulate the direction and effectiveness of the immune response in a way that is unfavorably for the organism. Since enterosorption has proven itself to be a fairly effective detoxifying means for many types of toxicosis (1–7), enterosorbents may help normalize not only intestinal microflora but also restore both antitumor and antimicrobial immunity through the detoxification mechanisms (**Figures 1**, **2**).

However, despite the seemingly evident expected positive effects of using enterosorbents in cancer treatment, further extensive preclinical and clinical studies are needed. These studies should account for different cancer types and localization and stages of the pathological process, gender and age differences, applied therapy courses, as well as the presence of gastroenterological diseases, and other characteristics of cancer patients. Moreover, in experimental preclinical studies, it would be highly beneficial to assess the comprehensive impact of enterosorption on the state of various components of the immune system arising from detoxification during the oncological process. Additionally, exploring the nature of the interaction between the microbiota, the immune system, and tumors in the dynamics of cancer development would provide a solid foundation for predicting the effect of enterosorption in clinical trials. It could aid in making informed decisions about prescribing enterosorption as an additional component of cancer therapy.

Understanding the intricate interplay between tumor growth, microbiota, immunity, and enterosorption is crucial for developing novel therapeutic approaches. Future research in this area may provide essential knowledge for optimizing enterosorption protocols that can enhance its efficacy in reducing immune tolerance to tumors, and ultimately improving the overall success of cancer treatment strategies.

## Author contributions

VS: Conceptualization, Data curation, Investigation, Resources, Visualization, Writing – original draft, Writing – review & editing.
